# Combined Transfer of Human VEGF165 and HGF Genes Renders Potent Angiogenic Effect in Ischemic Skeletal Muscle

**DOI:** 10.1371/journal.pone.0038776

**Published:** 2012-06-13

**Authors:** Pavel Makarevich, Zoya Tsokolaeva, Alexander Shevelev, Igor Rybalkin, Evgeny Shevchenko, Irina Beloglazova, Tatyana Vlasik, Vsevolod Tkachuk, Yelena Parfyonova

**Affiliations:** 1 Institute of Experimental Cardiology, Russian Cardiology Research and Production Complex, Moscow, Russia; 2 Department of Biological and Medical Chemistry, Faculty of Fundamental Medicine, Lomonosov Moscow State University, Moscow, Russia; 3 MonA LLC, Moscow, Russia; Bristol Heart Institute, University of Bristol, United Kingdom

## Abstract

Increased interest in development of combined gene therapy emerges from results of recent clinical trials that indicate good safety yet unexpected low efficacy of “single-gene” administration. Multiple studies showed that vascular endothelial growth factor 165 aminoacid form (VEGF165) and hepatocyte growth factor (HGF) can be used for induction of angiogenesis in ischemic myocardium and skeletal muscle. Gene transfer system composed of a novel cytomegalovirus-based (CMV) plasmid vector and codon-optimized human VEGF165 and HGF genes combined with intramuscular low-voltage electroporation was developed and tested *in vitro* and *in vivo*. Studies in HEK293T cell culture, murine skeletal muscle explants and ELISA of tissue homogenates showed efficacy of constructed plasmids. Functional activity of angiogenic proteins secreted by HEK293T after transfection by induction of tube formation in human umbilical vein endothelial cell (HUVEC) culture. HUVEC cells were used for *in vitro* experiments to assay the putative signaling pathways to be responsible for combined administration effect one of which could be the ERK1/2 pathway. *In vivo* tests of VEGF165 and HGF genes co-transfer were conceived in mouse model of hind limb ischemia. Intramuscular administration of plasmid encoding either VEGF165 or HGF gene resulted in increased perfusion compared to empty vector administration. Mice injected with a mixture of two plasmids (VEGF165+HGF) showed significant increase in perfusion compared to single plasmid injection. These findings were supported by increased CD31+ capillary and SMA+ vessel density in animals that received combined VEGF165 and HGF gene therapy compared to single gene therapy. Results of the study suggest that co-transfer of VEGF and HGF genes renders a robust angiogenic effect in ischemic skeletal muscle and may present interest as a potential therapeutic combination for treatment of ischemic disorders.

## Introduction

Therapeutic angiogenesis using plasmid vectors carrying growth factor gene is a broadly studied approach which has already entered clinical trials and remained an important field of translation research in recent decades. For example angiogenic potential of plasmids with VEGF, HGF and basic fibroblast growth factor has been shown previously [1]–[3]. Feasibility and safety of plasmid-mediated stimulation of angiogenesis makes it a very attractive choice for wide clinical application yet obtained data indicates that use of single angiogenic growth factor may be insufficient to render long-term positive effect in patients with ischemic disorders. Double-blind, placebo-controlled clinical trials of plasmid-based gene therapy in patients with peripheral artery disease showed its safety and moderate efficacy which is definitely below expectations [4], [5]. These results raise the problem of new approaches to increase beneficial impact of therapeutic angiogenesis questioning (1) factor(s) chosen for induction of angiogenesis, (2) vectors and their administration routes and (3) possibility to combine several genes.

Human studies showed safety of local administration of plasmid DNA [6], [7], however, in most cases authors reported extremely low transfection efficiency [8], [9]. The latter is a significant obstacle for clinical implication which can be partially surmounted by use of different additional methods – electroporation, ultrasound-mediated gene transfer, polymer protection etc [10]. Another possible solution is development of novel effective and safe plasmid vectors with higher gene expression capacity which upon delivery would induce robust therapeutic factor production. Several plasmid systems have been developed and tested for animal and human use [11]. In present study we constructed and used a novel plasmid vector which comprises CMV immediate early promoter and some other commonly used regulatory elements combined to increase protein yield.

Finally it is possible to enhance angiogenic response using gene therapy with physiologically additive combinations of factors. Published animal studies report increase of efficacy after gene therapy with pairs of VEGF/angiopoietin-1 [12], platelet-derived growth factor BB/fibroblast growth factor-2 [13] etc. Our previous experience in this field shows that delivery of VEGF165 and urokinase genes is an effective way to induce angiogenesis in ischemic skeletal muscle [14].

**Figure 1:**
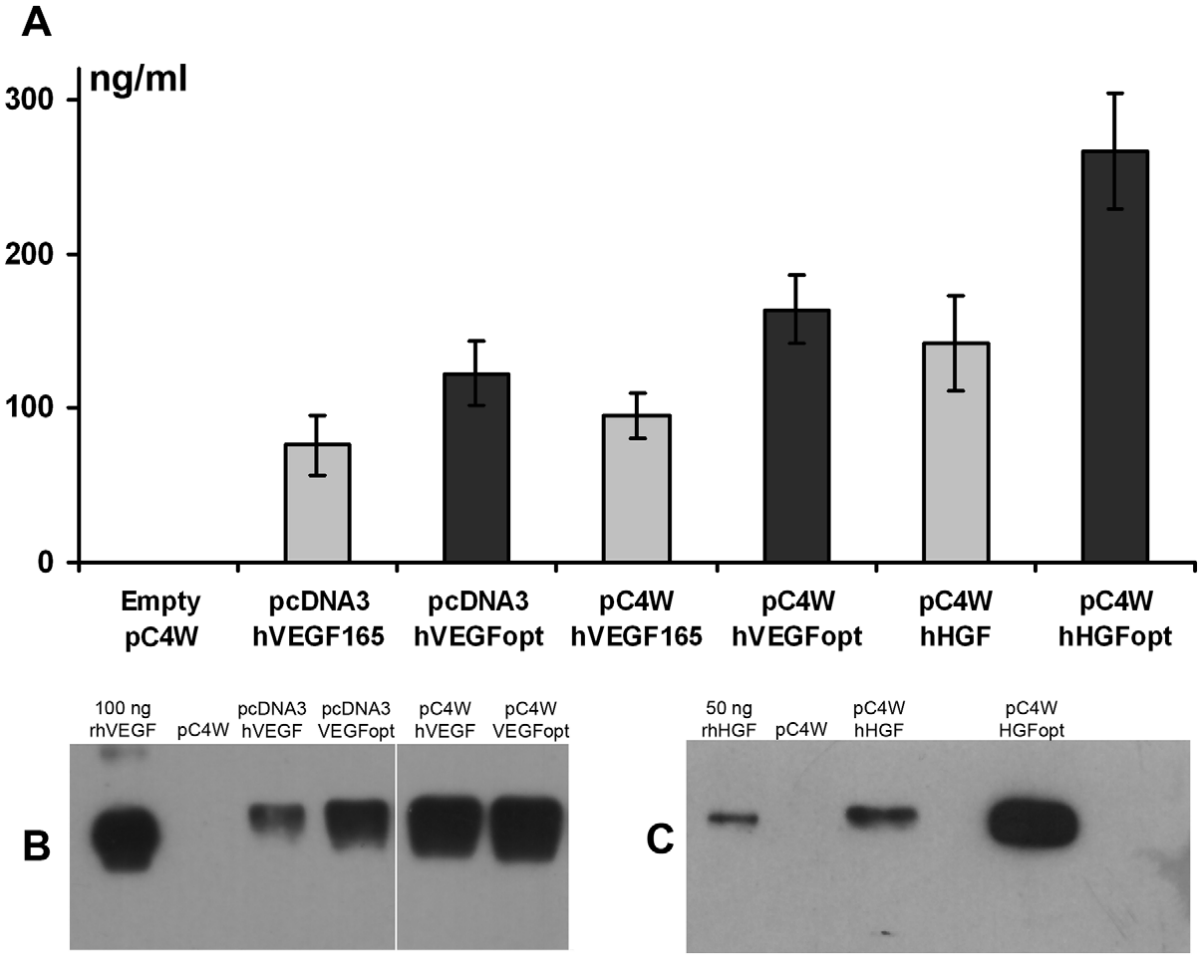
VEGF165 and HGF contents in condition medium collected from HEK293T after transfection with pcDNA3- or pC4W-based constructs with unoptimized/optimized cDNA sequence. (**A**) Human VEGF165 and HGF were assayed in conditioned medium collected 48h after transfection of HEK293T by pC4W or pcDNA3-based vectors. (**B**) and (**C**) – corresponding western blot radiograms of same medium samples after staining with antibodies against VEGF165 or HGF.

Among other growth factors a pair of VEGF and HGF is a potential candidate for therapeutic application. Both proteins are known to show morphogenic, mytogenic and antiapoptotic properties which are crucial for their regenerative potential [15]–[17]. Regulation of expression and mutual effects of VEGF and HGF has been thoroughly studied by many groups [18], [19] to identify possible basis for vivid *in vitro* findings of increased angiogenic response induced by combination of these two factors in cell culture and non-ischemic animal models of angiogenesis [20], [21]. Primarily angiogenic properties of HGF were attributed to its stimulating paracrine effect on VEGF production by endothelium. Further Van Belle et al. suggested that HGF effect was related to induction of VEGF secretion by smooth muscle cells [21]. However, later works clearly demonstrated that HGF and VEGF have independent effects [22] and distinct signaling pathways which mediate their activity via two tyrosine-kinase receptors – c-met and VEGFR2 respectively.

**Figure 2:**
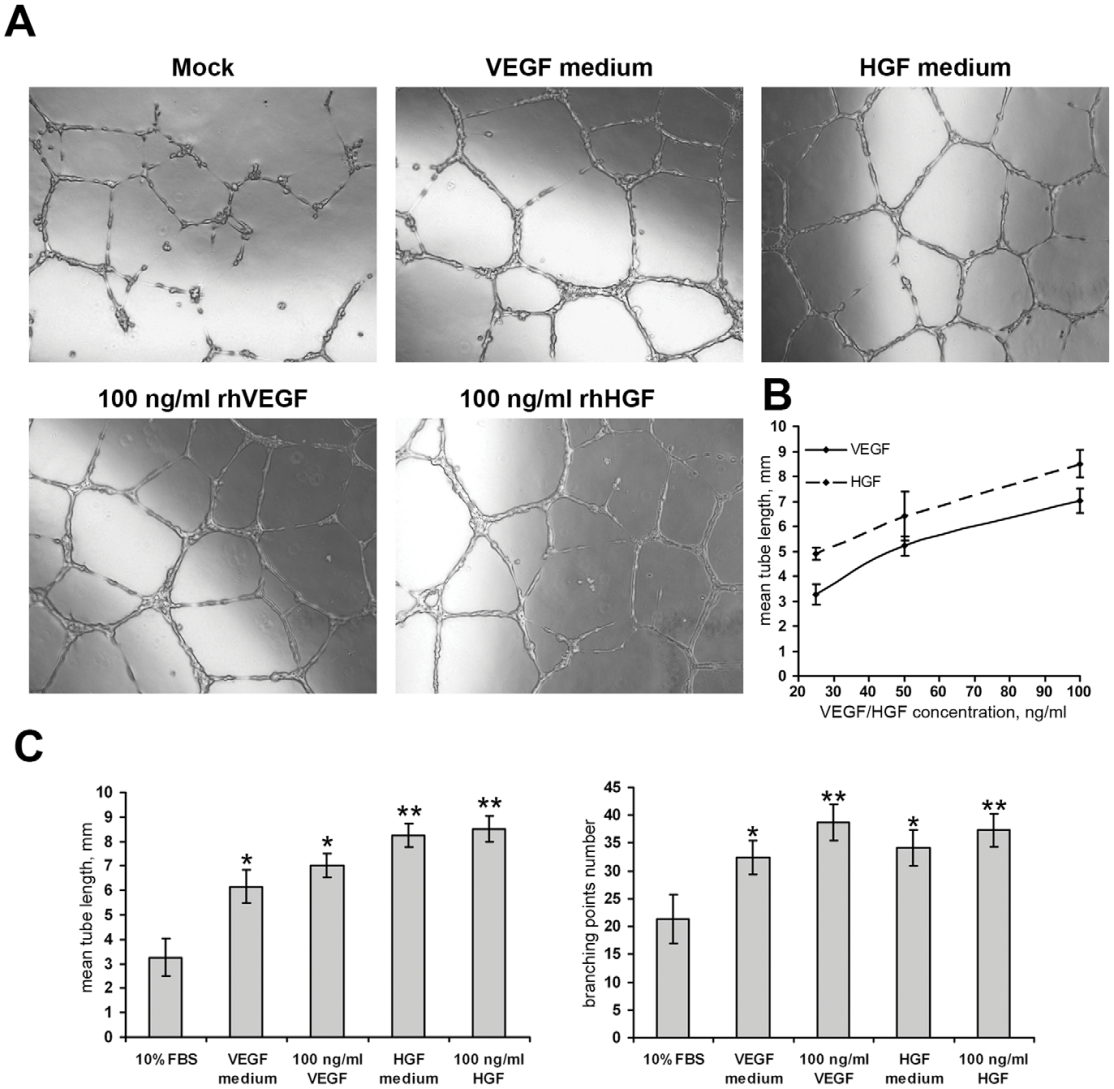
VEGF and HGF produced by transfected HEK293T cells induce distinct tubular structures formation in the HUVEC cell culture. (**A**) Tube formation in the HUVEC culture after incubation with conditioned media collected from transfected HEK293T cells or with positive control substance (100 ng/ml rhVEGF or rhHGF); 100x magnification, phase contrast. (**B**) Dose-response curves are obtained from wells with 25 ng/ml, 50 ng/ml or 100 ng/ml of recombinant human VEGF or HGF. Data of 3 serial experiments is presented. (**C**) Mean tube length (mm) and branching point number after HUVEC incubation with experimental conditioned media or positive control. For detailed data see Table 1.

Basing on these findings we hypothesized that combined transfer of VEGF and HGF genes to ischemic tissue could be a potent way to induce revascularization and restore blood flow. To demonstrate that we used a novel highly effective plasmid vector pC4W previously produced and described by our group [23] and intramuscular low-voltage electroporation to enhance gene delivery. We also performed codon optimization of chosen genes to increase their expression after plasmid transfection.

## Materials and Methods

### Ethics statement

All animal experiments carried out for the study were conducted in accordance with Institute of Experimental Cardiology guidelines. Animal procedures were approved by the Ethics Board of Institutional Animal Care and Use committee of Cardiology Research Complex (permit number 385.06.2009).

### Reagents and antibodies

#### Cell culture reagents

Dulbecco's modified Eagle medium (DMEM), M199 medium with Earle's salt, 100x antibiotic/antimycotic solution were purchased from Gibco, fetal bovine serum was purchased from HyClone. Growth-factor reduced Matrigel basement membrane matrix (Cat#354635), recombinant human VEGF165 (Cat#354107) and HGF (Cat#354103) were purchased from BD Biosciences.

**Table 1:** Tube formation assay results after incubation of HUVEC with 25 ng/ml rhVEGF, rhHGF or their combination.

	Tube length, mm	p vs neg. control	Branching points, n	p vs neg. control
**VEGF cond. medium**	6.16±0.6	0.03	32.4±3.1	0.009
**HGF cond. medium**	8.257±0.2	0.01	34.1±3.2	0.002
**rhVEGF, 100 ng/ml**	7.02±0.5	0.02	38.7±3.2	0.001
**rhHGF, 100 ng/ml**	8.51±0.5	0.01	37.7±2.9	0.01
**Negative control (10 % FBS)**	3.25±0.7	-	21.3±4.3	-

#### Plasmid purification

EndoFree plasmid Giga kit (Cat#12391) was purchased from Qiagen.

#### Elisa kits

human Quantikine® VEGF165 (DVE00) and HGF (DHG00) kits were purchased from R&D Systems.

#### Antibodies

mouse anti human VEGF165 monoclonal antibodies (Cat#MAB3045), mouse anti human HGF monoclonal antibodies (Cat#MAB294) were purchased from R&D Systems; Pharmingen rat anti mouse PECAM (CD31) monoclonal antibodies (Cat#550274) were purchased from BD Biosciences. FITC-labeled mouse polyclonal anti smooth muscle antibodies (Cat#F3777) were purchased from Sigma-Aldrich; AlexaFluor®594-conjugated donkey anti rat secondary antibodies (A21209) were purchased from Invitrogen; western blotting HRP-conjugated goat anti-mouse secondary antibodies (Cat#115-035-174) were purchased from Jackson Immunoresearch.

#### General reagents

Protease inhibitor cocktail (Cat#P-2714) was purchased from Sigma-Aldrich; 1x Quick Start Bradford Dye Reagent was purchased from Bio-Rad (Cat#500-0205);

**Figure 3:**
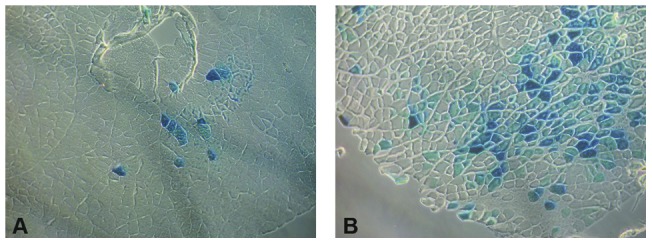
Histochemical detection of β-galactosidase activity in murine muscle. Representative images of X-Gal stained murine skeletal muscle cross sections after (**A**) Routine intramuscular injection of pC4W-βGAL plasmid (**B**) Injection followed by transcutaneous low-voltage pulses 50× magnification, phase contrast mode.

### Cell lines

Human embryonic kidney (HEK) 293T cells were obtained from ATCC; human umbilical vein endothelial cells (HUVEC) were kindly provided by prof. A.V. Mazurov DSc from Laboratory of cell adhesion of Institute of Experimental Cardiology [24].

### Animals

Eight-week old C57BL/6 male mice were used for hind limb ischemia experiments, *ex vivo* detection of transgene expression and electrotransfer efficacy evaluation with β-gal reporter plasmid. All animals received standard food and water ratios. Surgical manipulations and euthanasia protocols were designed in accordance to Institute and National regulations.

**Figure 4:**
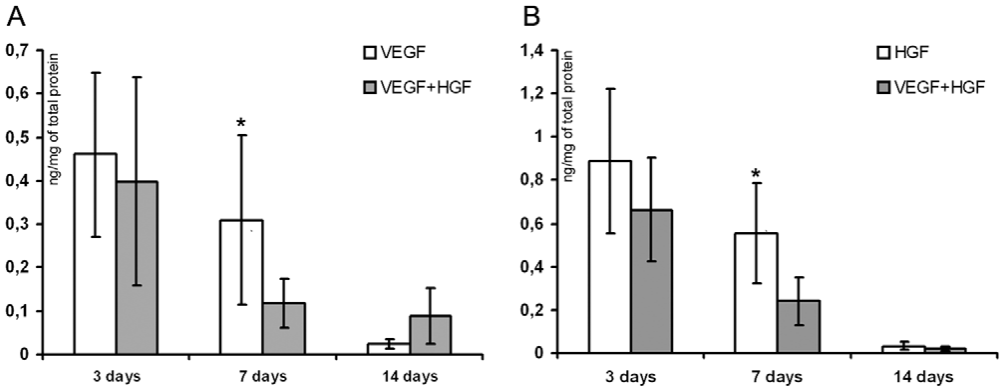
VEGF and HGF concentration in animal tissue after delivery of VEGF, HGF or VEGF+HGF to ischemic muscle. (**A**) and (**B**) – ELISA of human VEGF or HGF in transfected murine muscle homogenate sample at days 3, 7 and 14 post injection of single plasmids or their combinations. Data is presented as mean±SD after normalization of produced transgene quantity per mg of total protein. Mean of 3–4 samples assayed by specific ELISA presented. * p<0.05 vs VEGF+HGF.

### Plasmid construction and purification

Mammalian expression vector pC4W as well as codon-optimized human VEGF165 and HGF genes have been described earlier [23]. Unmodified and codon-optimized versions of each gene were subcloned into pcDNA3 (Invitrogen) and pC4W expression vectors for comparison. Reporter plasmid pC4W-βGAL was generated by subcloning the *E. Coli* β-galactosidase gene sequence into pC4W vector.

All plasmids were amplified in *Escherichia coli* (XL-blue) grown in LB medium and purified using EndoFree Plasmid Giga Kit (Qiagen). Standard LAL-test with aliquots of plasmids was performed to assay pyrogenicity of each formulation. In all tested samples endotoxin level did not exceed 10 EU per 1 mg of plasmid DNA, which complies to manufacturer’s range and existing Institute regulations for *in vitro* and animal tests.

**Figure 5:**
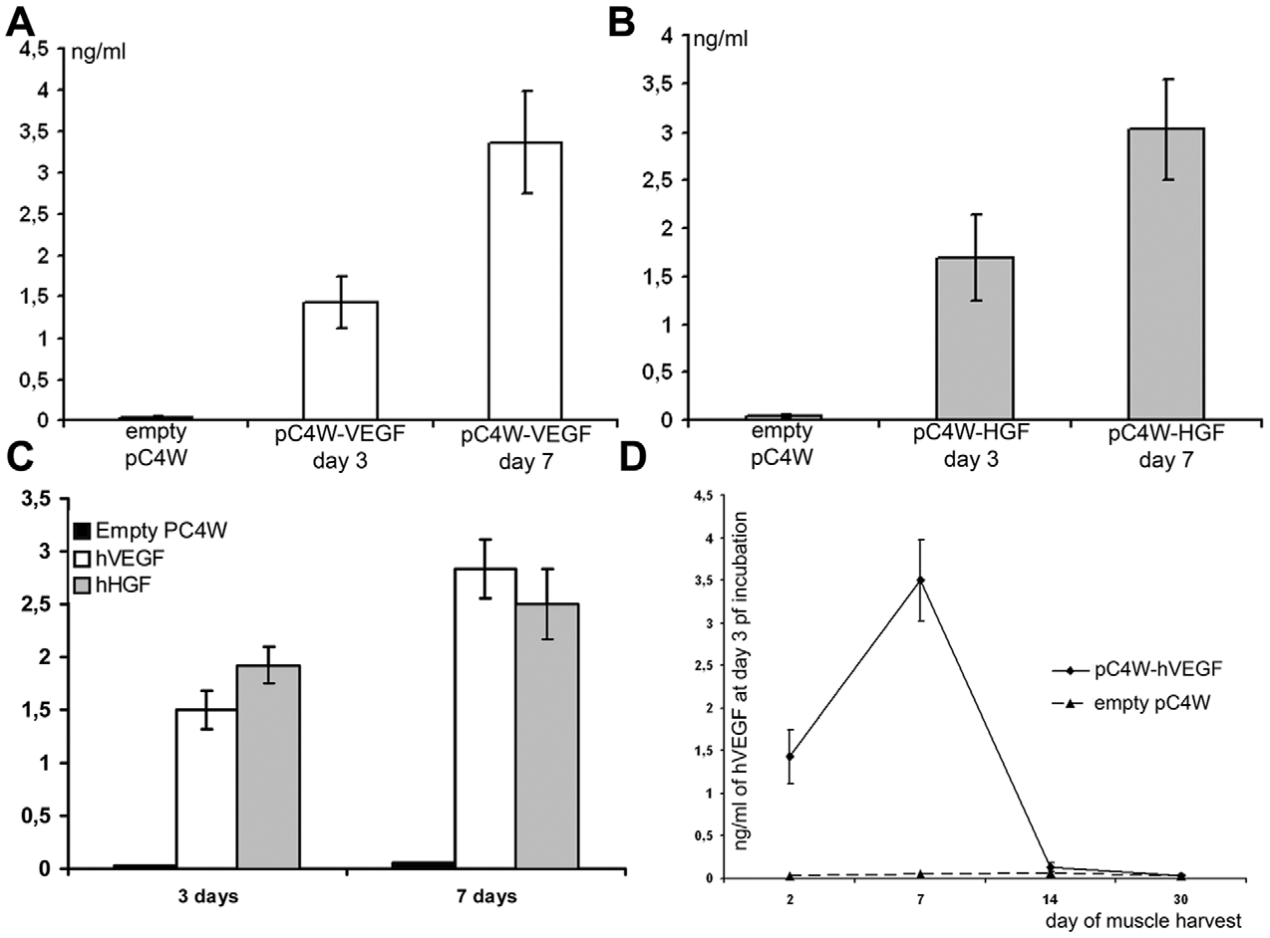
VEGF and HGF secretion by explanted muscle after *in vivo* gene transfer. (**A**) ELISA of human VEGF in medium from muscle explants prepared 2 days after 100 μg of pC4W-hVEGFopt plasmid or pC4W empty vector injection. (**B**) Analogous data for human HGF secretion after pC4W-hHGFopt plasmid injection. (**C**) Human VEGF and HGF secretion by muscle explants after a mixture of 100 μg pC4W-hVEGFopt and 100 μg pC4W-hHGFopt plasmid injection. (**D**) Dynamic profile of human VEGF production by trasnfected muscle. Explants harvested at different time points after VEGF plasmid injection were used to assay VEGF secretion.

### Cell culture and Ca^2+^/phosphate transfection

HEK293T cells were grown in complete DMEM. Transfection was performed on a 6-well plate at 80–85% confluent in 2 ml of complete DMEM. 0.2 ml of 2xHBS (50 mM HEPES, 280 mM NaCl, 1.5 mM Na_2_HPO_4_, pH = 7.1) was added by drops to 0.2 ml of 0.3 M CaCl_2_ mixed with 2 μg of pDNA. Resulted mixture was added to a 6-well plate followed by gentle swirling. Cells were incubated overnight under standard conditions and medium was replaced with condition medium (1% FBS DMEM). Aliquots of condition medium were collected 48 hours later, mixed with protease inhibitor cocktail and frozen at −70°C.

### Tube formation assay

Early passage (<P4) HUVEC were plated on Matrigel-coated 24-well dish in a mixture of 20% FBS HUVEC medium and low-serum conditioned medium from transfected HEK293T cells in 1∶1 ratio to obtain final FBS concentration of 10% (total volume 200 μl). Negative control cells were treated with a mock mixture of 20% FBS HUVEC medium and conditioned medium from empty pC4W-transfected HEK293T. Positive control wells were treated with 25, 50 or 100 ng/ml of rhVEGF165 or rhHGF added to 10% FBS HUVEC medium. After 16 hours of incubation under standard conditions tube formation was evaluated in microphotographs taken under 100× magnification in phase-contrast mode. Tube length and branching points numbers were counted in MetaMorph software in 5 random FOV taken from each plate well and mean values for control substance or tested medium sample were obtained. All samples were tested in duplicate within every experiment and total of 3 serial runs were taken for final evaluation.

**Figure 6:**
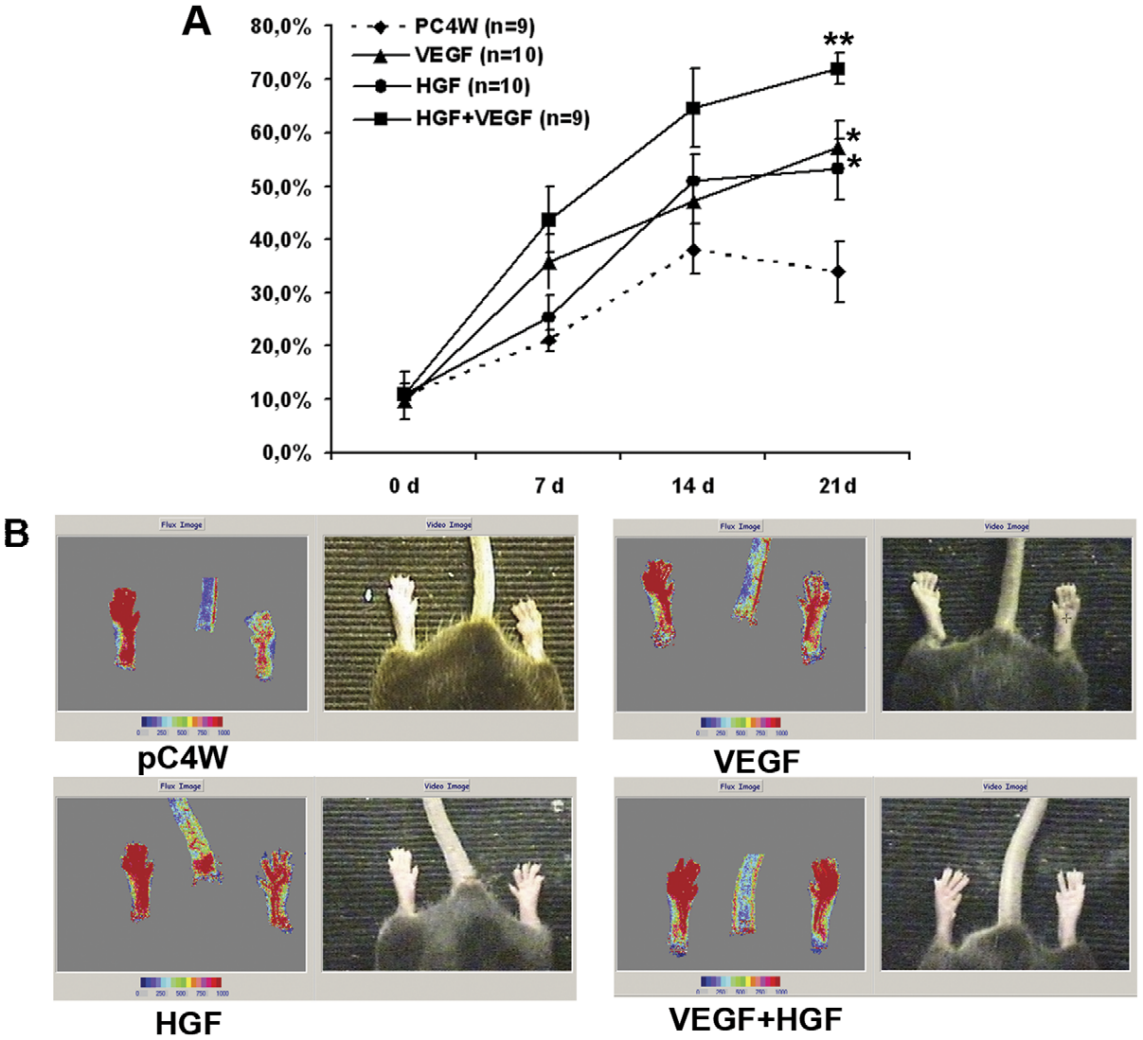
Laser Doppler imaging of ischemic limb reperfusion after growth factor gene transfer. (**A**) Dynamics of hind limb perfusion in experimental groups of mice that received pC4W empty vector, phVEGFopt (VEGF), phHGFopt (HGF) or combination of phVEGFopt and phHGFopt. Group details presented in text. * – p<0.05 vs pC4W, ** – p<0.05 vs pC4W and sole plasmids. (**B**) Representative laser Doppler images of the hind limb perfusion in experimental groups at the study endpoint (day 21).

### *Ex vivo* analysis of VEGF and HGF production by explanted muscle

Skeletal muscle explant culture experiments were performed in accordance with protocol described by Jang and Kim [25].

### Skeletal muscle disruption and human VEGF and HGF detection by ELISA

Skeletal muscle samples from animals injected with phHGF, phVEGF or their combination were harvested at day 3, 7 or 14 and used for transgene protein detection by human-specific ELISA (Quantikine®, R&D Systems). Negative control animals received empty vector injection. Briefly, animal was sacrificed and *m. tibialis anterior* was excised and washed in 1x PBS and then placed to a mortar pre-cooled on liquid nitrogen. After addition of 500 μl protein extraction buffer (0.5 M NaCl, 20 mM Tris (pH 7.5), 1 mM EDTA and 1x protease inhibitor cocktail) muscle was disrupted on liquid nitrogen with a pestle; pulverized sample was collected to a tube and then thawed on water bath at 37°C. Debris was removed by centrifugation (19000 g, 10 minutes) and cleared sample was used for Bradford total protein assay and ELISA detection of human growth factors. For total protein assay the samples were diluted up to 20 fold using protein extraction buffer, for ELISA up to 5 times in accordance to manufacturer's protocol.

**Figure 7:**
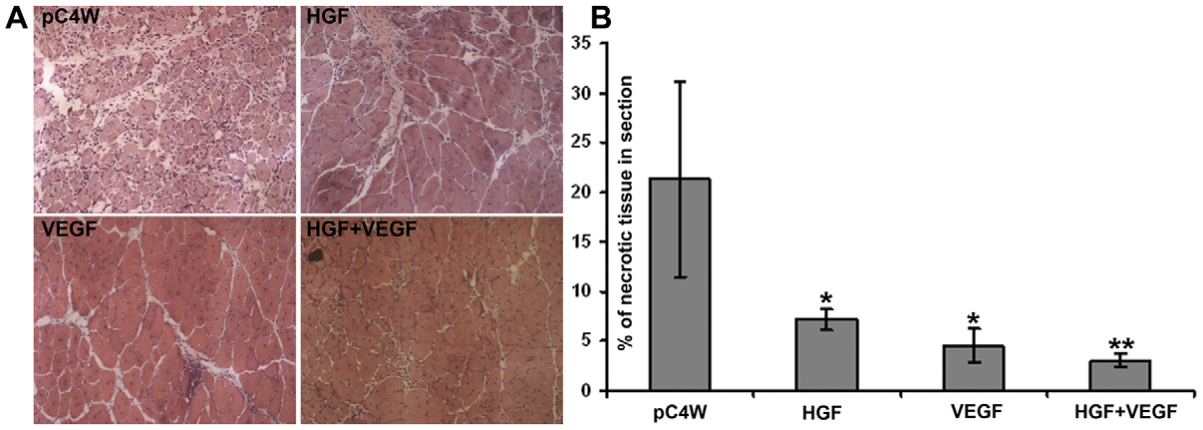
Histological analysis of necrosis in muscle samples from study group animals. (**A**) Representative images of muscle sections from different study groups. 200× magnification, hematoxylin-eosin staining. (**B**) Mean necrosis area/total section area for study groups. *p<0.05 vs pC4W; **p<0.05 vs VEGF or HGF.

### ELISA of human VEGF and HGF

We used manufacturer's protocol (R&D Systems) for ELISA detection of VEGF165 and HGF in medium samples and tissue homogenates by corresponding Quantikine® kit.

### Western blotting

Samples of culture medium were used for SDS-denaturing electrophoresis in a polyacrilamide gel under non-reducing conditions according to standard procedures. Separated proteins were transferred to a PVDF membrane (Millipore) with subsequent staining by monoclonal mouse anti human antibodies against VEGF165 or HGF overnight at 4°C and with secondary polyclonal HRP-conjugated goat anti mouse IgG antibodies for 1 hour at room temperature. Two-component West Pico chemiluminescent substrate system (Pierce) was used for development of secondary antibodies.

**Figure 8:**
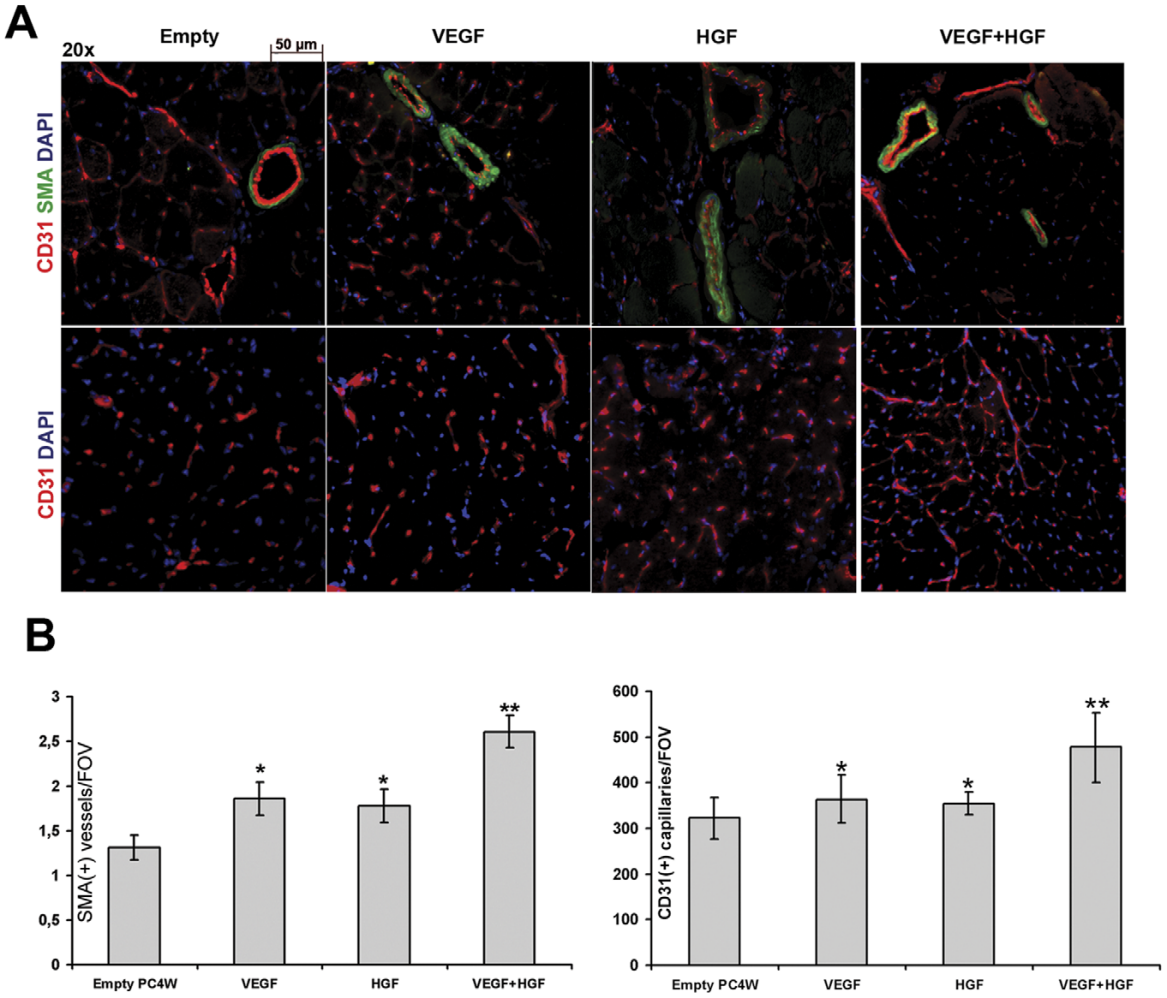
Histological analysis of murine ischemic skeletal muscle neovascularization. (**A**) Representative images of sections stained for smooth muscle actin and CD31. (**B**) SMA+ and CD31+ vessel counts per field of view. *p<0.05 vs pC4W; **p<0.05 vs pC4W-VEGF or HGF.

**Table 2:** Capillary (CD31) and arteriole (SMA) density in muscle sections from different study groups.

	SMA+ arteriolae	p vs control (Mann-Whitney U)	CD31+ capillaries	p vs control (Student's t)
**Empty pC4W**	1.31±0.15	-	322.4±44.9	-
**VEGF**	1.86±0.19	0.03	364.2±53.0	0.0001
**HGF**	1.78±0.21	0.002	354.6±25.0	0.002
**VEGF+HGF**	2.6±0.18	0.001	477.7±75.6	0.0001

### Mouse hind limb ischemia model and injection of plasmid solutions

Mice were narcotized by intraperitoneal injection of 2.5% avertin solution calculated by body mass. Unilateral induction of hind limb ischemia was performed as previously described [26]. All surgical manipulations were carried out in aseptic conditions under binocular microscope. Briefly, skin was incised along midline of left hind limb hip and *a. femoralis* with its branches was ligated distal to inguinal ligament and proximal to its popliteal bifurcation. Vessel was excised between upper and lower ligatures and after control of hemostasis skin was closed with 5–0 silk sutures. After completion of surgery animals were put until full recovery into a chamber on an ambient heated pad. After surgery one of plasmid formulations was injected to *m. tibialis anterior* of ischemic limb. Animals were randomized to receive 100 μg pC4W-hVEGFopt (n = 10) or 100 μg pC4W-hHGFopt (n = 10). Combined transfer group received a mixture of plasmids: 100 μg pC4W-hHGFopt+100 μg pC4W-hVEGFopt (n = 9). Negative controls were injected with 100 μg of empty pC4W vector (n = 9). All injected plasmids (including combined transfer) were diluted in 100 μl of sterile saline throughout all study groups. Injection was followed by transcutaneous electric pulses as previously described by Schertzer and Lynch [27] with a minor modification – we omitted injection of hyalouronidase to muscle. For electric pulsing we used BTX-Harvard apparatus ECM 830 square wave pulse generator equipped with tweezer electrodes.

**Figure 9:**
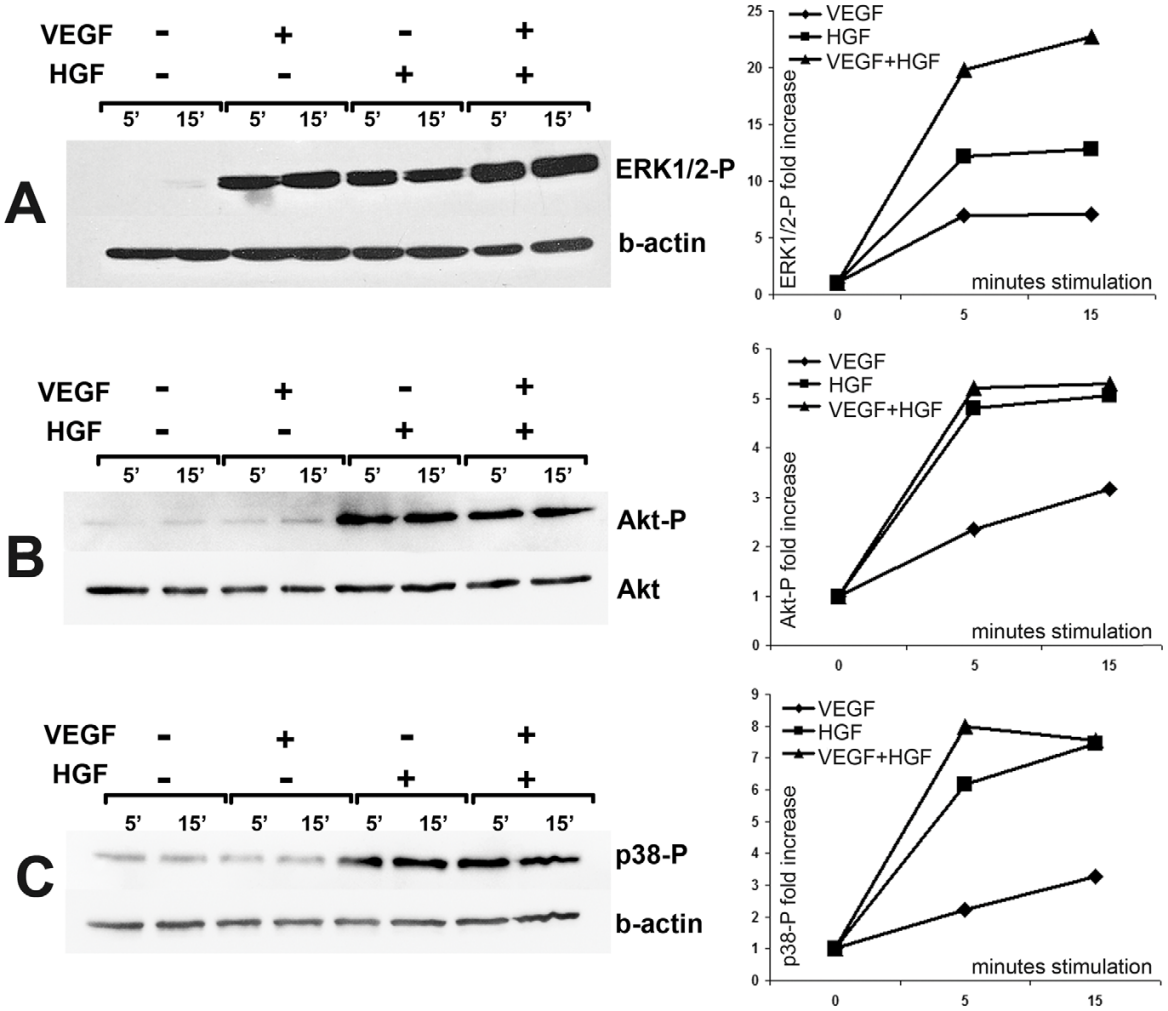
VEGF and HGF cooperate to induce ERK1/2, but not Akt or P38-mediated intracellular signaling. (**A–C**) – HUVEC were stimulated by 25 ng/ml of VEGF165, HGF or their combination (5 or 15 minutes). Cell lysates were analyzed using western blot with specific antibodies against phosphorylated ERK1/2, Akt or P38 and normalized against β-actin or Akt to obtain densitometric data and corresponding graphs (right column).

### Laser Doppler perfusion measurement

Blood perfusion was assessed using a Moor LDI 2.0 system in isoflurane-narcotized animals at 7-day intervals for 3 weeks. Animals were placed to an induction chamber, narcotized by isoflurane inhalation and then placed on an ambient heating pad for 10 minutes under 1–2% isoflurane/oxygen inhalation for stabilization of narcosis depth and blood pressure. Consequent perfusion measurements on plantar surface of animal's feet were made and data variability was analyzed using Moor image review software. Readings were taken until 3 subsequent runs with minimal (<10%) deviation were obtained. To account for variability among measurements, ambient light and temperature fluctuations all raw perfusion units readings were normalized against non-ischemic limb and expressed as relative perfusion per cent.

### Muscle harvest and histological analysis

At day 21 animals from test groups were sacrificed by lethal isoflurane dose followed by cervical dislocation and ischemic *m. tibialis anterior* was harvested and frozen in TissueTek medium. Serial frozen sections (7 μm) were prepared and stained for tissue analysis.

We used hematoxylin-eosin staining for tissue necrosis analysis. Necrotic muscle span is expressed as percent to total section area. Signs of muscle necrosis were loss of fiber morphology, cytoplasm disruption, inflammatory cells infiltration and fibrosis.

For immunofluorescent analysis sections were formalin-fixed and then incubated overnight with corresponding primary and – after wash – secondary antibodies (2 h) and counterstained with DAPI. Stained sections were mounted in aqeous-based medium for subsequent microscopy.

Microphotographs of sections were taken under 200× magnification in 5 random FOV per section using Zeiss Axiovert 200 M fluorescent microscope with Axiovision 3.1 software. Manual vessel counts in microphotographs were made by two independent persons in MetaMorph 7.1.0.0 software. Capillary density analysis included manual counts of CD31-positive structures per FOV; arteriolar counts were obtained as number of SMA(+) blood vessels with clearly visible CD31(+) inner layer per FOV. Capillary and arteriole counts per FOV were used to obtain mean values for section, animal or group and were subject to subsequent statistical assessment.

### X-gal staining of tissue sections

Frozen section of muscle were formalin-fixed and stained for β-galactosidase activity by incubating them for 24 hours in warm PBS-based solution containing 5 mM potassium ferricyanide crystalline; 5 mM potassium ferricyanide trihydrate; 2 mM MgCl_2_ and 1 mg/ml X-Gal (Sigma-Aldrich). Slides were incubated under 37°C overnight and then washed in PBS and mounted in aqueous-based medium for further microscopy. Transfection efficacy was expressed as ratio of β-galactosidase positive muscle fibers/total fibers in section.

### Statistical analysis

Data is expressed as mean ± SD (SEM in some exceptions). Statistical significance of difference between 2 groups was determined using a Student's t-test or Mann-Whitney rank sum U-test depending on sample distribution profile analyzed by Shapiro-Wilk test. Multiple groups were compared using appropriate statistical method with Bonferroni's correction for critical level of significance. Statsoft Statistica 6.0 was used for analyses of study data.

## Results

### Plasmid constructs encoding codon-optimized human VEGF165 and HGF genes

We have constructed a novel mammalian expression vector pC4W. Briefly the expression cassette of pC4W consists of human CMV immediate-early promoter/enhancer, rabbit beta-globin intervening sequence-2, consensus translation initiation sequence, woodchuck hepatitis virus posttranscriptional regulatory element (WPRE), and bovine growth hormone and simian virus 40 early polyadenylation signals.

To enhance the expression of VEGF and HGF genes we used codon optimization strategy based on the genetic code redundancy. Coding regions of human VEGF165 and HGF gene were screened for the presence of codons occurring with the lowest frequency in human open reading frames. Using conventional genetic engineering and gene synthesis technologies, rare codons were replaced by the most frequent codons encoding the same amino acids, resulting in preservation of the amino-acid sequence of each protein. Furthermore, two signals of mRNA polyadenylation AATAAA and two mRNA destabilizing signals ATTTA [28] were excised from the human HGF coding region using same technique.

### Study of VEGF165 and HGF expression in cell culture

To assay expression activity of different plasmid constructs *in vitro* we transfected them to HEK293T cells. Aliquots of condition medium collected after transfection were assayed for human VEGF165 or HGF contents by Western blotting and ELISA. As shown in Fig. 1 (B, C) cells treated with pC4W-based constructs produce more target protein compared to cells transfected with pcDNA3-based constructs encoding the same gene. Furthermore, application of plasmid constructs with optimized genes hVEGFopt and hHGFopt resulted in higher protein production than after transfection by plasmids encoding unmodified VEGF or HGF sequences. Quantitative ELISA of conditioned media confirmed that use of pC4W-based vectors with optimized cDNA sequences resulted in highest VEGF or HGF production (Fig. 1, A). According to this all further *ex vivo* and animal experiments were carried out using plasmids with the highest protein yield in HEK293T cell culture – pC4W-hVEGFopt and pC4W-hHGFopt.

### Secreted VEGF165 and HGF induce tube formation in HUVEC culture

To evaluate angiogenic activity of VEGF and HGF produced by HEK293T after transfection with pC4W-hVEGFopt and pC4W-hHGFopt we collected medium containing secreted proteins and transferred it to human umbilical vein endothelial cells (HUVEC) on Matrigel-coated surface. Freshly isolated HUVEC were plated in a mixture of double (20%) FBS HUVEC medium and conditioned medium from transfected HEK293T cells in 1∶1 ratio. Tube formation after 12–14 hours of incubation was evaluated by measuring mean tube length and branching point (BP) number per field of view at 100× magnification.

As expected, HUVEC showed comparable angiogenic response after treatment with 100 ng/ml of positive control proteins (rhVEGF or rhHGF); for details see Table 1 and Fig. 2. In HUVEC incubated with medium from VEGF- or HGF-expressing HEK293T mean tube length and BP counts were significantly higher than in negative control and were comparable to positive control stimulations with VEGF and HGF.

### Low-voltage electroporation enhances plasmid gene transfer to murine skeletal muscle

To assay effect of low-voltage pulses on gene delivery after single intramuscular injection of plasmid DNA we used pC4W-based β-galactosidase expressing reporter construct. It was injected to *m. tibialis anterior* and transcutaneous pulses were applied as described [27]. Muscles were harvested 3 days later and frozen sections were prepared and stained by X-Gal to detect β-galactosidase activity (Fig. 3). β-galactosidase positive fibers percentage was 17.1±4.7% after electroporation vs. 2.2±1.2% without electroporation (p<0.001). Our data demonstrates that low-voltage pulses leads to dramatic increase of β-galactosidase positive fibers number and, thus, overall transfection efficacy.

### Production of human VEGF165 and HGF by murine skeletal muscle

Production of human growth factors in murine muscle after gene transfer was detected by ELISA of tissue samples and medium samples collected from muscle explant cultures. Homogenates were prepared from muscle samples taken at day 3, 7 and 14 post injection (pC4W-hVEGFopt, pC4WhHGFopt or their 1∶1 mixture) and after centrifugation were assayed with a human-specific kit (R&D Systems).

Significant amount of transgenic protein (normalized to total protein amount) was detectable in samples taken at every time-point we used yet declining tendency can be obviously seen in presented data (Fig. 4 A, B). No cross-reactivity was found and signal in negative control (empty vector injection) or cross-group analyses was below limits of detection.

Muscle explants were prepared from *m. tibialis anterior* harvested 2 days after injection of plasmid DNA (VEGF, HGF or combination). At days 3 and 7 after explantation medium samples were collected for ELISA of human VEGF or HGF contents. Single VEGF or HGF plasmid injection followed by low-voltage electroporation resulted in increasing over time concentration of human angiogenic factor in medium that contained skeletal muscle explant (Fig. 5, A, B). Very little background signal was detected in control explants injected with empty pC4W vector.

In our homogenate ELISA tests we found significant gene expression timespan of at least 14 days after injection of single plasmid or their mixture (Fig. 4). We also analyzed human VEGF secretion by muscle explants form animals sacrificed at days 2, 7, 14 and 30 after VEGF plasmid injection. Human VEGF production was maximum in samples taken at day 7 after injection and then gradually decreased being still detectable in samples taken at day 14 after phVEGF administration (Fig. 5, C) with almost no signal at day 30 indicating gene expression fadeout during week 3.

Expression of human VEGF and HGF after combined gene transfer was assayed using both models. Mice were injected with a mixture of 100 μg phVEGF and 100 μg phHGF plasmids and after a period of time muscle samples were harvested and used for ELISA or explanted in Matrigel. In explant culture of a muscle harvested at day 2 after injection we found that amount of each angiogenic factor after plasmid mixture administration was comparable to values obtained after injection of sole plasmid encoding the corresponding human gene (Fig. 5, D). Direct ELISA of muscle homogenates taken at day 3 showed comparable data yet in later terms (at day 7) we found a significant increment in human growth factor contents in sole plasmid groups compared to VEGF+HGF which still diminished by day 14 as total production of transgenic factor declined (Fig 4).

### Effect of human VEGF and HGF co-transfer in mouse hind limb ischemia model

All animals had similar perfusion rates before and after induction of ischemia and no significant deviation of baseline values at day 0 were found within groups. After induction of unilateral ischemia mice were distributed to experimental groups and received one of the following pro-angiogenic gene therapy treatments:

1. pC4W-hVEGFopt (100 μg in 100 μl of saline).
2. pC4W-hHGFopr (100 μg in 100 μl of saline).
3. mixture of pC4W-hVEGFopt and pC4W-hHGFopr (100 μg each in total volume of 100 μl).
4. pC4W empty vector as negative control (100 μg in 100 μl of saline).

Limb perfusion measurements were performed at days 7, 14 and 21 after surgery and plasmid injection as described in Materials and Methods. No significant necrotic changes were observed in most animals throughout the study, yet in some control mice amputations of toes were registered.

We found that single injection of 100 μg pC4W-hVEGFopt (VEGF group) or pC4W-hHGFopt (HGF group) plasmids followed by electroporation rendered significant and comparable angiogenic effect by day 21 showing increase of perfusion compared to the empty vector pC4W injection (Fig. 6). Relative perfusion values in animals that were subjected to combined (VEGF + HGF) gene transfer were higher than in any other group.

As for combined VEGF and HGF gene transfer we injected 200 μg of total plasmid DNA versus 100 μg for single gene transfer, we also assessed the influence of plasmid DNA quantity on angiogenic effect expressed as restoration of perfusion. For that purpose we conducted additional animal test with double dose of pC4W-hVEGFopt plasmid. By day 21 after surgery and plasmid administration mice that received 200 μg of pC4W-hVEGFopt plasmid showed perfusion rates similar to mice injected with 100 μg of the same plasmid (data not shown).

### Histological analysis of skeletal muscle

At day 21 after surgery and plasmid administration animals were sacrificed and frozen cross-sections of *m. tibialis anterior* were prepared for further histological analysis.

Necrosis signs were especially prominent in sections of the central portion of *m. tibialis anterior*. Disrupted tissue span expressed as necrotic tissue area/total area section was significantly higher in empty vector group than in any other experimental group indicating severe ischemic damage to skeletal muscle. Ischemic muscle disruption was drastically reduced in animals treated with VEGF, HGF plasmid or their combination (Fig. 7).

CD31-positive (CD31+) capillaries and smooth muscle actin positive (SMA+) vessels were counted after double immunfluorescent staining (Fig. 8) to provide morphological evidence of endothelial proliferation and mature vessel formation after gene transfer. CD31+ capillary density as well as SMA+ vessel number increase was registered in animals injected with single VEGF or HGF plasmid compared to empty vector group. In combined gene transfer group (VEGF + HGF) we found significantly higher capillary density and SMA+ vessel number compared to VEGF or HGF groups (Table 2) indicating higher sprouting activity in capillary endothelium cells induced by administration of two angiogenic growth factors.

### VEGF and HGF cooperatively stimulate ERK1/2 phosphorylation

To investigate which signalling pathways are activated by combination of VEGF and HGF we analyzed phosphorylation of several MAPKs (mitogen activated protein kinase) in HUVEC stimulated by recombinant HGF, VEGF or both. VEGFR-2 and c-met downstream cascades may have several cross-talk points and using western blot with antibodies specific against phosphorylated forms of ERK1/2 (extracellular-signal-regulated kinase 1/2), p38 kinase or Akt we found that treatment with VEGF and HGF together resulted in greater phosphorylation of ERK1/2. In our study we didn’t observe enhanced activation of Akt and p38 after VEGF+HGF addition and found HGF to be a more potent inductor of Akt and P38 (Fig. 9). As for single factor stimulation we used 25 ng/ml of VEGF or HGF and for co-stimulation we used amount of proteins to create 25 ng/ml of each factor (50 ng/ml total protein) we also analyzed ERK1/2 activation in 50 ng/ml VEGF or HGF. This test intended to show that increment in ERK1/2 phosphorylation was not due to higher amount of protein in cell medium. We didn’t find increase of ERK1/2 activation after doubling VEGF or HGF concentration and same data was obtained for p38 and Akt. Effect over time curves showed slight difference between VEGF and HGF property to activate ERK1/2 yet stimulation by VEGF+HGF led to stable enhancing effect during the period of stimulation.

## Discussion

Limb ischemia is a great medico-social burden which along with population ageing that correlates with decrease of body regenerative capacity [29] will emphasize this problem in the future and drive new investigations in this field.

Completed and ongoing clinical trials focus on single growth factor administration for induction of angiogenesis. Most of them show moderate to low beneficial effect of angiogenic “monotherapy” for peripheral artery disease after pDNA injection to impaired tissue. Since the original successful attempt by J. Isner [1] gene therapy of limb ischemia was a field of great promise, but results of first placebo-controlled clinical trials became a real disappointment for most specialists [30]. Clinical studies of VEGF165 plasmid showed it safety but low efficacy in most cases [4], [31]. Recent trials with HGF showed its safety yet insignificant beneficial effects in critical limb ischemia [32], [33] and moderate efficacy in Buerger’s disease [2]. Finally, recent failure of a phase III trial of NV1FGF [34] became another proof that field of therapeutic angiogenesis requires new possible ways to increase efficacy and novel approaches for gene delivery. One of concepts that is intensively studied in recent decade is to apply combined gene therapy by several growth factors with independent targets/receptors and pleiotropic beneficial properties. Thus, development of *in vivo* applicable vectors is also a great point especially taking into consideration that plasmid vectors are safe and feasible with minimal immunogenicity (compared to viral particles) which makes them an attractive object for implication in combined transfer.

In our study we tested a new delivery tool – a plasmid vector with high protein output and used a combination of two growth factors – VEGF165 and HGF to augment angiogenesis in ischemic skeletal muscle.

VEGF165 is a well-known growth factor and has become an important object of biological and medical research due to its role in angiogenesis, tumor growth and development. For deeper insights into its biological properties one can be advised to pay attention to a series of excellent reviews by N. Ferrara et al. [17], [35]–[37]. Therapeutic use of VEGF165 became a major object for clinical implication yet many studies report mediocre to low efficacy and significant dose-limiting side effects: tissue edema, inflammatory response due to adhesion molecule upregulation etc. The second of a pair – HGF is known to activate endothelial cells migration, proliferation and tube formation although in contrast to VEGF these effects are not limited to endothelial cells. HGF induction is observed in injured skeletal muscle and myocardium and in many tumors and tumor-derived cell lines along with its receptor – c-met. Important feature of HGF for therapeutic angiogenesis is its ability to induce significant angiogenic response without increasing vascular permeability or inducing inflammatory changes in the tissue [38].

In previous studies combination of HGF and VEGF165 has been tested in endothelial cell cultures. *In vitro* application of HGF+VEGF results in a more robust proliferative and chemotactic response than each growth factor alone. More complicated 3D collagen models showed that only combination of VEGF and HGF was capable of inducing signification tubulogenic response and facilitate cell survival, yet neither VEGF nor HGF alone was [20]. Studies by Min et al. showed that in HUVEC culture HGF can inhibit VEGF-induced expression of adhesion molecules ICAM-1 and VCAM-1 suppressing inflammatory response both in cell culture and in animal hypersensitivity reaction model. This effect was attributed to HGF ability to inhibit NF-kB pathway activated by VEGFR2 downstream signaling [39]. These findings show created a basis for further *in vivo* studies using rhHGF and rhVEGF in rabbit ischemic limb and corneal angiogenesis assay to support previous findings [21], but combination of VEGF165 and HGF has never been tested in gene transfer studies.

In first part of our work we tested a novel plasmid vector and evaluated its efficacy compared to conventional pcDNA3 vector system. Novel pC4W vector consists of CMV promoter and a number of additional regulatory elements which are combined to obtain higher protein yield. In our cell culture tests we found higher target protein contents after transfection with pC4W-based plasmids compared to pcDNA3-based ones. We also showed additional expression enhancement obtained by codon optimization of angiogenic factor genes selected for our study. Using more effective vectors combined with a safe enhancing technique (e.g. electrotransfer we applied) may result in more successful angiogenesis induction. It seems that there can be a “threshold” concentration/gradient of growth factor which is required to induce cell proliferation and migration leading to formation of a vessel or to promote survival of both – pre-existing and dividing endothelial cells. Thus if transfected tissue generates higher amount of therapeutic protein this can lead to better tissue protection against ischemia preventing necrosis, fibrosis or pathological proliferation. In our work we used explant model and tissue homogenate ELISA to confirm human protein secretion by transfected murine muscle. This allowed us to detect secreted human transgene and to ensure that vector activity is sufficient to provide significant amount of target proteins.

Regarding combined angiogenic factors gene transfer little is known about administration of several plasmids in one “shot”. Our data indicates that in early terms (2–3 days after injection) protein production after combined delivery is similar to single gene transfer. However, ELISA tests of muscle homogenate samples show that at approximately 1 week there is a significant difference in production of a “couple” of factors by transfected muscle compared to single factor delivery. Tissue VEGF and HGF concentration after single gene delivery were higher than after combined plasmid injection. This finding that creates an image of discrepancy at first glance makes increase in perfusion we observed after VEGF+HGF treatment even more intriguing. It can point out a possible case for biological amplification of effect as total “therapeutic protein” amount is constant yet perfusion is enhanced. Thus, our data can indicate that chosen therapeutic factors in lesser quantities can deliver greater benefit when acting together over a period of time after delivery to ischemic tissue. Regarding later time-points we found that by day 14 both proteins are secreted in comparably low quantities and sole plasmid groups have the same protein yield as combined transfer. Similar data was obtained by Korpisalo et al [40] for adenoviral delivery of VEGF-A and PDGF-B to ischemic muscle, yet very few observations are made in regard to plasmid-mediated co-delivery of 2 therapeutic proteins. Still while analyzing obtained data we should take into consideration that direct tissue sample analysis reflects *in situ* protein production and explant cultures are a great tool for long-term detection of transgene (especially when its production declines) due to protein accumulation in condition medium over time.

We evaluated expression period of pC4W-based vector in explants and tissue samples harvested at different time-points after injection. Protein production was maximum in specimen taken at days 3–7 with major decline in samples taken at day 14 reflecting drastic fall of protein production. Plasmid vector activity decrement after 2 weeks since administration to skeletal muscle is attributed to plasmid DNA efflux and degradation resulting in lower copy number and subsequent protein extinction. This “fading period” during the 2^nd^ week might be the most appropriate time point for administration of a repeated injection of plasmid DNA to support angiogenic response and factor concentration.

*In vivo* tests were performed in mice with unilateral limb ischemia. VEGF- and HGF-induced limb reperfusion after plasmid injection has been reported and reproduced in many works and has been translated to clinical trials. In our study efficient stimulation of angiogenesis after injection of constructed vectors to ischemic skeletal muscle was shown both by blood flow increase and histological findings which included higher CD31+ and SMA+ vessel counts reflecting dense capillary network formation and arteriogenic properties of both factors. Novel finding in this experiment is increasing (up to 75%) perfusion after injection of VEGF+HGF plasmids mixture. We found perfusion in combined VEGF+HGF group to be the highest compared to single plasmid or control. Laser Doppler data was supported by higher capillary and arteriolar density found in combined transfer group animals compared to sole HGF or VEGF groups.

Our *in vitro* tests in HUVEC cultures indicate that one of putative pathways responsible for angiogenic effect amplification could the ERK1/2 signaling cascade. In our studies we didn’t find increase in Akt or p38 phosphorylation yet in all cases HGF was a more potent inductor of studied pathways than VEGF165. Thus, we can speculate that one of possible mechanisms for amplified angiogenic response is ERK1/2-mediated increase of endothelium proliferation. Most published data covers several possible mechanisms of interaction including thorough study of signaling cross-talk yet comprehensive explanation is still elusive and will be the subject of further studies.

Thus, to conclude, our data indicates that novel pC4W vector is a highly effective and feasible plasmid for therapeutic gene delivery to skeletal muscle. *Ex vivo* experiments showed that gene of interest expression retains for at least 14 days after single administration of pDNA. Further we also showed that angio- and arteriogenesis in skeletal muscle can be successfully induced and augmented by administration of combination of pC4W-based plasmids with optimized cDNAs of VEGF165 and HGF.

Summarizing our data we can expect the combination of VEGF and HGF to become one of candidates for future translational research along with other candidate pairs: VEGF+angiopoietin-1, VEGF+FGF e t c.

By the moment all published observations regarding use of several growth factors gene transfer are limited to animal studies due to safety reasons but after first pilot trials and accumulation of safety and prospective data we can expect this approach to become a “new level” technique in therapeutic angiogenesis. Translation of this method to clinical practice may also require development of next generation bicistronic vectors suitable for human use to administer two genes in one plasmid but use of two separate vectors for reach gene suggested in our study seems to have a great advantage of equal expression of both therapeutic factors which popular IRES-based bicistronic vectors lack after *in vivo* administration as they do *in vitro*.
